# Static and Dynamic Properties of Semi-Crystalline Polyethylene

**DOI:** 10.3390/polym8040077

**Published:** 2016-03-28

**Authors:** Ming-ming Xu, Guang-yan Huang, Shun-shan Feng, Graham J. McShane, William J. Stronge

**Affiliations:** 1 State Key Laboratory of Explosion Science and Technology, Beijing Institute of Technology, Beijing 100081, China; mintg922@bit.edu.cn (M.X.); ssfeng@bit.edu.cn (S.F.); 2 Department of Engineering, University of Cambridge, Cambridge CB2 1PZ, UK; wjs3@cam.ac.uk

**Keywords:** extruded polyethylene, plastic flow, molecular structure, mechanical behavior, split Hopkinson bar

## Abstract

Properties of extruded polymers are strongly affected by molecular structure. For two different semi-crystalline polymers, low-density polyethylene (LDPE) and ultra-high molecular weight polyethylene (UHMWPE), this investigation measures the elastic modulus, plastic flow stress and strain-rate dependence of yield stress. Also, it examines the effect of molecular structure on post-necking tensile fracture. The static and dynamic material tests reveal that extruded UHMWPE has a somewhat larger yield stress and much larger strain to failure than LDPE. For both types of polyethylene, the strain at tensile failure decreases with increasing strain-rate. For strain-rates 0.001–3400 s^−1^, the yield stress variation is accurately represented by the Cowper–Symonds equation. These results indicate that, at high strain rates, UHMWPE is more energy absorbent than LDPE as a result of its long chain molecular structure with few branches.

## 1. Introduction

**Figure 1:**
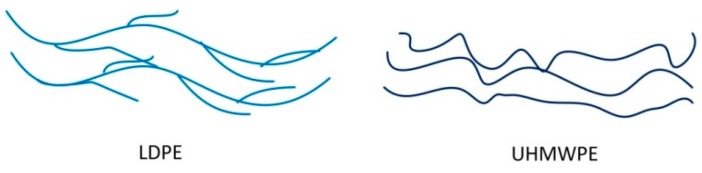
Schematic molecular structure representation of two types of extruded polyethylene.

For use in a light laminated armor, extruded polyethylene is a lightweight, low cost material with medium strength and high ductility. Recent theoretical and experimental assessments of metal-polymer bilayers indicate significant potential for increasing the ballistic limit velocity in applications where lightweight is an advantage [1]. This is especially the case for polymer layers which show significant strain-hardening [2,3]. Both static and dynamic mechanical properties of extruded polymers are strongly affected by their molecular structure. For ultra-high molecular weight polyethylene (UHMWPE), Kurz *et al.* [4,5] presented an isotropic, small strain exponential model that fit the true stress-strain data (true strain < 0.12). Pouriayevali *et al.* [6] and Epee *et al.* [7] proposed a constitutive model to describe the quasi-static and high strain-rate large deformation response of semi-crystalline polymers at strain-rates < 800 s^−1^. These experimental data and theories precisely describe mechanical response and viscoelasticity at a small strain, but they are not helpful for characterizing the behavior for large plastic deformation that precedes fracture. Further characterization of large, dynamic deformation behavior of semi-crystalline polyethylene is required. In this paper, the mechanical properties of two semi-crystalline polyethylenes, low density polyethylene (LDPE) and UHMWPE, were measured in order to investigate the effect of molecular structure on properties at large strains and high strain-rates [8,9,10] (see Figure 1). The aim is to determine those mechanical properties that are most important for resisting projectile penetration and perforation of bilayer metal-polymer laminates.

In the present investigation, quasi-static tension and compression material tests were conducted on specimens cut from polyethylene plate at three different angles (0°, 45° and 90°) relative to the extrusion direction. The static tests were performed in an INSTRON universal testing machine till fracture, which gave strain rates of 0.001 and 0.01 s^−1^. Because early necking in tension tests will result in nonhomogeneous deformation field in post-necking tensile, an optical technique was used for strain measurements. In addition, dynamic impact tests of LDPE and UHMWPE were carried out using a split Hopkinson pressure bar at room temperature; this gave strain rates of 680~3300 s^−1^ which are similar to those that occur in projectile impact tests. Typical mechanical response of specimens are compared and discussed with regard to the effect of molecular structure on mechanical properties such as anisotropy, yield, strain-hardening, microstructure evolution, strain-rate sensitivity and capability for energy absorption. Furthermore, the high strain-rate data has been fitted to the Cowper–Symonds model of yield stress dependence on strain rate. This investigation provides a useful experimental database for future dynamic penetration research.

## 2. Materials and Methods

### 2.1. Materials and Experimental Program

LDPE and UHMWPE are both low density, semi-crystalline, thermoplastic polymers but for UHMWPE the molecular weight (4 × 10^6^–6 × 10^6^) is much larger than that of LDPE (0.028 × 10^6^–0.28 × 10^6^) [11,12]. These polyethylenes have a similar density of molecular chains but the length of the chains is much longer in UHMWPE [12]. Extruded plates of LDPE and UHMWPE were obtained from the plastic extruding machine. The PE powders were introduced into the feed inlet port of the extruder that used 150 °C for LDPE and 220 °C for UHMWPE with a screw rotation speed of 75 rpm. The mixtures were compression-molded into the extruded rolls (320 mm in diameter with a width of 1000 mm) with a laboratory press. The materials were cooled down to 23 °C by using a room-temperature water circulation within the press plates. Finally, the PE plates were cut into 3 mm thickness square plates with 500 mm edge length. Properties of these materials, such as density *ρ*, Young’s modulus *E*, yield stress σ*_y_*, strain at break ε and vicat softening point *T*, provided by the manufacturer are presented in Table 1. There are several reasons for this choice of materials: their similar low density (0.91 g/cm^3^ for extruded LDPE and 0.95 g/cm^3^ for extruded UHMWPE), mid-range strength and relatively low cost. Consequently, these polymers are similar except for molecular weight (which is related to the length of polymer chains). The effect of this difference in molecular structure on static and dynamic properties of polyethylene will be examined in terms of yield strength, rate sensitivity, strain hardening modulus, *etc.*

### 2.2. Quasi-Static Tensile and Compressive Tests

**Figure 2:**
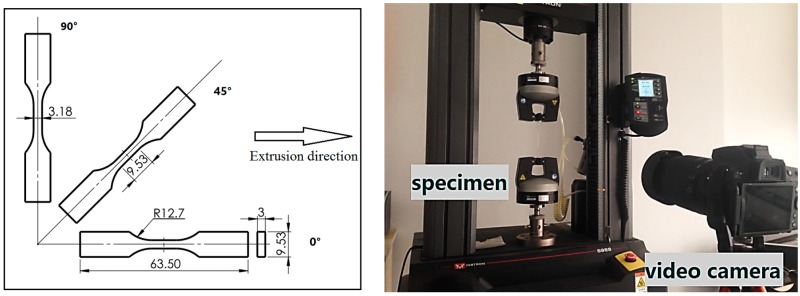
Geometry of tension specimen and the tensile test set-up with video camera.

Specimens for tensile tests were dog-bone shaped based on the ASTM D638-03 type V specification while specimens for compression tests were cylinders with dimensions of 10 mm in diameter by 10 mm in length. All specimens were machined from extruded plates and tested to failure on an INSTRON-5969 (INSTRON, Norwood, MA, USA) universal testing machine at room temperature 23 °C and 50% relative humidity condition. Five specimens were tested at the same strain rate for each sample group in order to calculate the mean value. It is well known that anisotropy can be an important characteristic for some extruded thermoplastics, especially in the extrusion direction. Therefore, to measure anisotropy in each polyethylene material, tension coupons oriented at three different angles (0°, 45° and 90°) with respect to the extrusion direction were cut from plates as shown in Figure 2. Thus, the specimens for strain rate 0.001 s^−1^ were divided into three groups of five specimens each for three angles. To measure large plastic deformation in the tensile test, a digital image correlation technique was adopted to obtain accurate stress-strain curves. In the tensile tests, a correlation technique was used to obtain strain measurements. Namely, two black points demarked the gauge region of each specimen and a video camera recorded the movement of the two points during each test, see Figure 2. Then an image-processing program (IMP) was applied to process these images of each specimen and subsequently acquire the strain-time curve using MATLAB.

**Figure 3:**
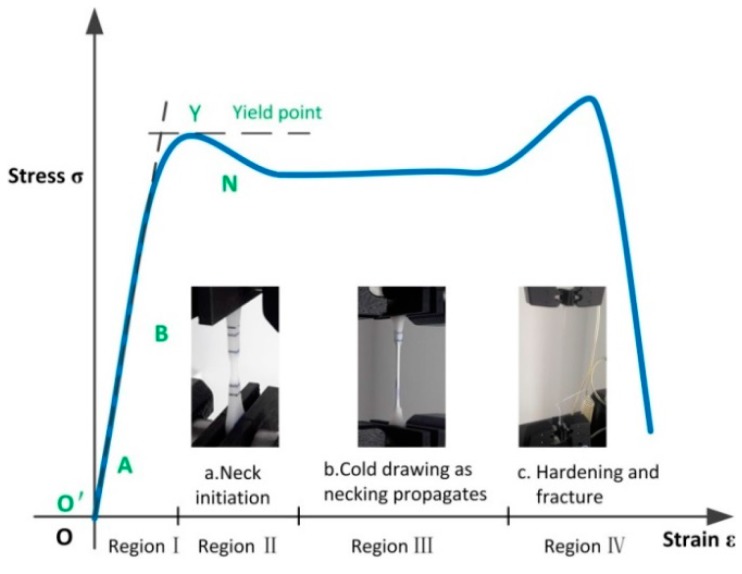
Typical engineering stress-strain curve of semi-crystalline polymer.

The polyethylene specimens for tensile tests were tensioned at constant crosshead speeds of 5.7 and 57 mm/min, respectively, corresponding to engineering strain-rates of 0.001 and 0.01 s^−1^. Although the formation of a neck during the cold drawing process will affect strain rate, the flow curves determined at constant true strain rate are similar to those determined at constant cross-head velocity [13]. The engineering strain-rate has been defined as ${\dot{\varepsilon }}_{t}=\frac{d\varepsilon }{\mathrm{dt}}=\frac{1}{L_{0}}\frac{d\dot{L}}{\mathrm{dt}}$ where *L* and *L*_0_ are the current length and gauge length [13]. The saddle shaped stress-strain curve shown in Figure 3 is typical of a test where necking develops within the gauge length; *i.e.*, the strain development occurs in a short, highly strained part of the gauge length and this necking region propagates along the specimen, away from some weak section where necking initiates.

Even for strains below the yield strain, the stress-strain relations of semi-crystalline polymer are nonlinear. For such materials, the slope of the tangent to the stress-strain curve at small stress is usually taken as the modulus of elasticity, as shown in Figure 3. Generally, this polymer stress-strain curve can be divided into four distinct regions—each representative of a specific phenomenon. This will be described in Section 3.1.2.

Compression tests of polyethylene specimens were performed under constant crosshead speeds of 6 and 60 mm/min, respectively, corresponding to strain rates of 0.001 and 0.01 s^−1^. Based on the assumption that the deformation is homogeneous or nearly homogeneous along the gauge length and that the specimen volume is constant, the relationship between nominal and true values of strain and stress can be expressed as [14]:

(1) $$\{\begin{array}{c} \varepsilon_{t}=\ln(1+\varepsilon_{n})\\ \sigma_{t}=\sigma_{n}(1+\varepsilon_{n}) \end{array},$$

where $\sigma_{t}$ and $\varepsilon_{t}$ are true stress and strain, $\sigma_{n}$ and $\varepsilon_{n}$ are nominal (engineering) stress and strain.

### 2.3. Dynamic Compression Test Apparatus (SHPB Tests)

**Figure 4:**
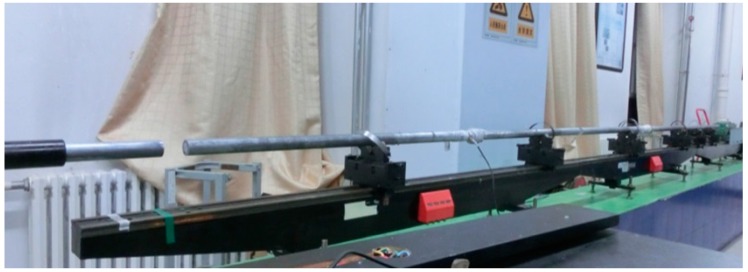
The compressive split-Hopkinson pressure bar (SHPB) system.

**Figure 5:**
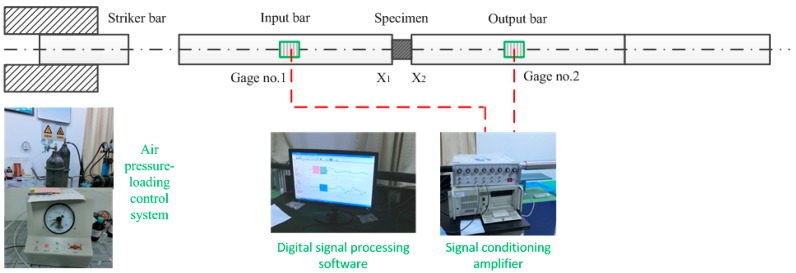
The schematic diagram of SHPB apparatus used in the experiment.

The dynamic tests were performed on an SHPB apparatus powered by a compressed-air gun (see Figure 4). The schematic diagram of the SHPB for compression testing is shown in Figure 5. It consists of three elastic pressure bars and a projectile, each with a diameter of 37 mm. The Aluminum bars had a Young’s modulus of 70 GPa. The lengths of incident bar, transmitter bar and buffer bar are 2 m, and the striker bar that is accelerated by air pressure has a length of 0.8 m. The gas pressure is controlled in order to provide an accurate and consistent striker bar speed. After the striker bar is propelled towards the input bar by the gas gun, the bars collide and an elastic compressive wave is generated by the impact. This compressive wave propagates backward through the input bar. The stress amplitude of incident impact wave $\sigma_{I}(=\rho C_{0}v/2)$ and the pulse duration $\Delta t(=2L_{0}/C_{0})$ can be adjusted by changing the impact velocity *v* and length *L*_0_ of the striker bar, respectively (*C*_0_ and ρ are the sound velocity and density of the Aluminum bars).

At the interface between the input bar and the specimen, part of the wave is reflected and part of the wave is transmitted through the specimen to the output bar. The reflected wave travels back as a tensile wave. The resulting time dependent strains ($\varepsilon_{I}(X_{G1})$, $\varepsilon_{R}(X_{G1})$ and $\varepsilon_{T}(X_{G2})$) are calculated from the voltage signals, measured by two strain gauges that are equidistant from the specimen; one attached to the input bar, for the incident and reflected signals and one attached to the output bar for the transmitted signal. This arrangement is shown in Figure 5. The SHPB test analysis is based on two assumptions [15]: (a) the propagation of the wave in the Aluminum bars was approximated by a one-dimensional theory, where the wave dispersion was negligible; (b) the stress and strain states in the specimen were homogeneous. Thus, the relationship between stress, strain-rate, and strain in the specimen, can be expressed as follows [16]:

(2) $$\sigma_{s}(t)=\frac{EA}{A_{s}}\varepsilon_{T}(X_{G2},t)=\frac{EA}{A_{s}}[\varepsilon_{I}(X_{G1},t)+\varepsilon_{R}(X_{G1},t)],$$

(3) $$\varepsilon_{s}(t)=\frac{2C_{0}}{l_{s}}\int_{0}^{t}\varepsilon_{R}(X_{G1},t)dt=\frac{2C_{0}}{l_{s}}\int_{0}^{t}[\varepsilon_{I}(X_{G1},t)-\varepsilon_{T}(X_{G2},t)]dt,$$

where *E, A* and *C*_0_ are the Young’s modulus, cross-sectional area, and wave velocity of the steel bars, respectively. $l_{s}$ and $A_{s}$ refer to the length and cross-sectional area of the sample. $\varepsilon_{I}(X_{G1})$, $\varepsilon_{R}(X_{G1})$ and $\varepsilon_{T}(X_{G2})$ are the recorded axial strains of the incident pulse, reflected pulse and transmitted pulse, respectively, measured in the input and output bars, as functions of time. A detailed analysis can be found in references [17,18].

Currently, there is no standardized specimen geometry for SHPB testing, so specimen design is a significant aspect of the research presented in this paper. As is well known, two requirements for SHPB specimens are a small gauge length to reduce ring-up time and inertial effects, as well as a suitable length to diameter ratio that results in a uniaxial stress state during pulse transmission [19].

To fit within the diameters of incident and output bars, the diameter of specimens of LDPE and UHMWPE were both 20 mm. Because the limitations on the driving gas pressure of SHPB equipment, the experiments adopted specimens having a range of thicknesses in order to obtain a wide range of strain rates. The specimens of LDPE and UHMWPE have thicknesses of 10, 5, and 4 mm, and these provide strain rates that are inversely proportional to the length of specimen. The specimen with a thickness of 10 mm when loaded by 152 kPa pressure achieves a low strain rate, 5 mm thick specimens loaded by either 152 or 304 kPa pressure respectively achieve a middle strain rate while 4 mm thick specimens loaded by 405 kPa pressure achieve a high strain rate. For the polyethylene specimens sandwiched between the input and output bars, the above pressures gave average strain rates of: 700, 1300, 2150, and 3300 s^−1^ (see Table 2).

## 3. Results and Discussion

### 3.1. Quasi-Static Uniaxial Tensile Tests

#### 3.1.1. Anisotropy of Extruded Polyethylene Materials

**Figure 6:**
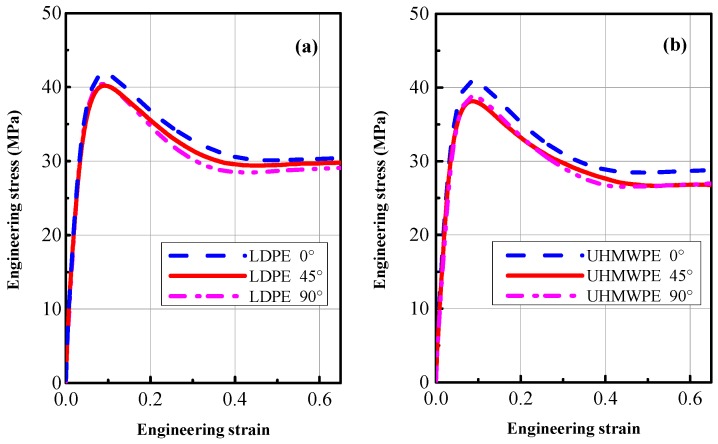
Engineering stress-strain curves of LDPE and UHMWPE at 0.001 s^−1^ strain rate for three directions relative to extrusion direction.

The main purpose of the tensile tests was to obtain comparative mechanical properties of these two extruded polyethylenes. In this investigation, all mechanical quasi-static properties of materials, including anisotropy, yield behavior, strength, and elongation at break, are summarized in Table 3. The quasi-static engineering stress-strain curves of LDPE and UHMWPE specimens oriented at three angles (0°, 45° and 90°) relative to the extrusion direction are shown in Figure 6. It can be seen that LDPE and UHMWPE show almost no anisotropy—the maximum difference between the stress-strain curves for the three angles is less than 3 MPa. The initial slopes of LDPE and UHMWPE, representing Young’s modulus E, show a slight difference depending on the orientation relative to the extrusion direction. The maximum difference of the average tensile strength for the three angles is 0.88 ± 0.37 MPa for LDPE and 2.76 ± 2.06 MPa for UHMWPE, which are insignificant in comparison with the tensile strengths. As these two types of extruded polyethylene cooled from the melt form, the molecular chains developed a disordered structure called the amorphous state—this is similar in all directions. Extrusion tends to cause some molecular orientation along the extruding direction. Nevertheless, the mechanical properties of the extruded polyethylene plate are roughly isotropic.

An important observation from Figure 6 is that the properties of LDPE and UHMWPE experience the same post-yield softening effect; *i.e.*, the decrease in stress to a constant flow stress is isotropic. This softening effect is probably connected to the evolution of volumetric strains caused by necking. Moreover, the constant plastic flow stress can be related to the molecular chain structure and distribution as will be explained on a macromolecular level based on the chemical reaction model presented by Zhurkov [20,21].

#### 3.1.2. Plastic Flow Characteristics under Tensile Loading

**Figure 7:**
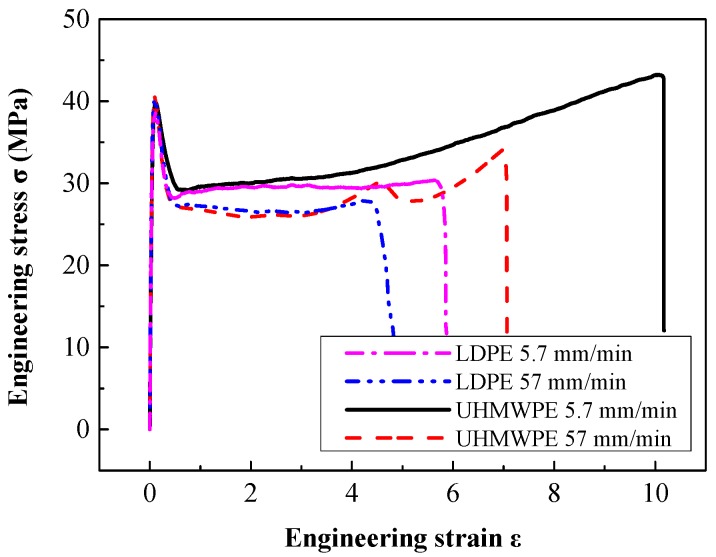
Tensile engineering stress-strain curves of LDPE and UHMWPE at 0° angle and strain rates of 0.001 s^−1^ (5.7 mm/min) and 0.01 s^−1^ (57 mm/min).

The typical engineering stress-strain curves of LDPE and UHMWPE at two strain rates and a range of strain that results in failure are shown in Figure 7. From the curves in this figure, we can see that the two types of polyethylene display similar engineering stress-strain curves, especially in the elastic region. Figure 7 shows that in a tensile test before necking starts, the stress rises in an approximately linear manner as the applied strain increases. After the peak stress, the nominal stress subsequently decreases during neck initiation in a small part of the gauge section as shown in Figure 3a. Typically, four distinct regions can be identified on the engineering stress-strain curves of these “ductile” polymers [8]: (1) elastic region; (2) initial necking region; (3) cold drawing region; (4) strain-hardening to fracture region, as shown in Figure 3. Molecular orientation and reorientation in macromolecular chains evolution under tensile force are unique inherent characteristics for polymer materials. The large fracture strain of LDPE and UHMWPE specimens at two strain rates in Figure 7 indicates a strong interior molecular chain orientation phenomenon which accompanies extension and slippage phenomena. In addition, the peak stresses (tensile strength, 40~41 MPa) at yield of the two polyethylene types are similar (experimental data summarized in Table 3). These results are similar because these polyethylenes have very similar distributions of molecular chain densities.

**Figure 8:**
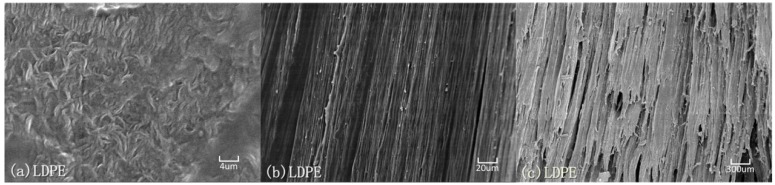
Electron micrographs of microstructure evolution in semi-crystalline LDPE under large plastic deformation: (**a**) original state, ε = 0; (**b**) highly oriented texture along the loading direction, ε = 1; (**c**) fracture surface, ε = 4.7.

**Figure 9:**
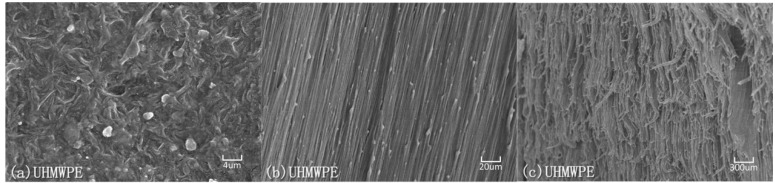
Electron micrographs of microstructure evolution in semi-crystalline UHMWPE under large plastic deformation: (**a**) original state, ε = 0; (**b**) highly oriented texture along the loading direction, ε = 1; (**c**) fracture surface, ε = 6.6.

Electron scanning micrographs (SEM) of LDPE and UHMWPE specimens are shown in Figure 8 and Figure 9. The specimens were sectioned parallel to the loading direction and the sectioned surface was sprayed with Pt film by sputter coater for visualization in the SEM. These micrographs visibly illustrate the distinct difference in molecular chain structure and distribution between LDPE and UHMWPE by comparing their distinguishable semi-crystalline organization images both in the undeformed state and on the fracture surface.

During tensile stretching in the constant flow stress region of Figure 7, the nominal stress is approximately constant for a considerable range of strain as the neck propagates along the length of the specimen—A phenomenon termed “stable necking” [13], as shown in Figure 3b. Meanwhile, continuous and constant stretching in the necking region produces both molecular orientation and small regions of three-dimensional order. In this plateau stress region for both LDPE and UHMWPE, the flow stress decreases with small but increasing strain-rates, being approximately 29 MPa and 27 MPa between strain-rates of 0.001 s^−1^ and 0.01 s^−1^, respectively.

Finally, in the strain-hardening to fracture region, when the propagating neck has spread over the whole of specimen length, further stretching causes the oriented molecular chains to be stretched until they break. Phenomenologically, the molecular orientation causes a diminution of the cross section near the center of the gauge section [22]. The fractographs in Figure 8 and Figure 9 for LDPE and UHMWPE exhibited the same typical ductile fracture pattern but there were striking differences in fracture appearance. At large strains both polymers became highly oriented under tensile loading as shown in Figure 8b and Figure 9b. However, the fracture appearance of LDPE in Figure 8c is flaky and inhomogeneous, which is quite different from UHMWPE’s homogeneous fracture surface in Figure 9c. Because molecular weight and the length of the molecular chain are internal factors of high molecular polymers, this affects their strength enhancement and tenacity. The shorter, more heavily branched molecular chains of extruded LDPE slipped and separated at a smaller strain than those of UHMWPE, leaving fewer molecular chains per unit area to bear the tensile force at large deformations. At large deformations, more molecular scission per unit area occur in extruded LDPE molecular links than occur in extruded UHMWPE at the same elongation. This decreases the effectiveness of strain-hardening and contributes to premature failure of extruded LDPE because the fiber bundles on the fracture surface are inhomogeneous and hackly as shown in Figure 8c. Quite different from the molecular chains of LDPE, the UHMWPE has long molecular chains and relatively little branching. These molecular chains can fully extend or stretch under tensile loading, making fracture of the fiber bundle both homogeneous and uniform after a large strain, as shown in Figure 8c.

#### 3.1.3. Fracture of Polyethylene

**Figure 10:**
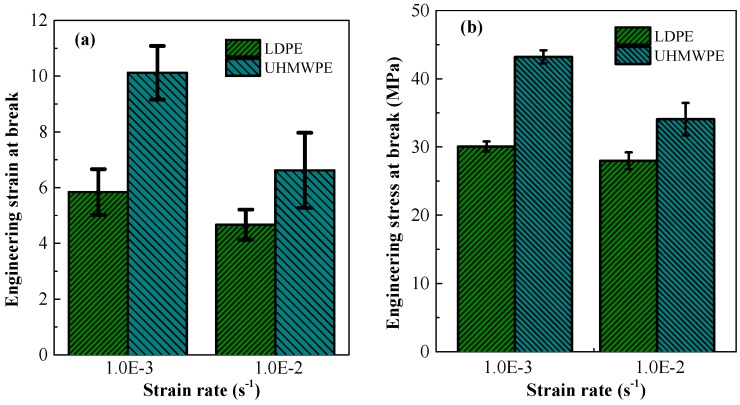
Contrasting engineering (**a**) strain and (**b**) stress at fracture of LDPE and UHMWPE at strain-rate 0.001 and 0.01 s^−1^.

In Figure 10a, the engineering strain at fracture of extruded UHMWPE is 10.12 at a strain-rate of 0.001 s^−1^—This was almost twice the strain at fracture of extruded LDPE. Moreover, in Figure 10b, the engineering fracture stress of extruded UHMWPE was larger than that of extruded LDPE at every strain rate. There was a strong strain-hardening effect for UHMWPE in the quasi-static tensile test, which resulted in a larger engineering stress and elongation at failure. Strain hardening improves the energy absorbing capacity of polyethylene.

Furthermore, fracture of polyethylene was greatly influenced by strain rate, as at high strain rates, the molecular chains were unable to coordinate and rapidly deform. When comparing the mechanical performance at tensile failure, the tensile properties of extruded UHMWPE were more sensitive to strain-rate. Increasing the strain-rate from 0.001 to 0.01 s^−1^ increased slightly the initial yield strength of extruded UHMWPE, as can be seen from the true stress-strain curves in Figure 3; however, this somewhat larger strain-rate significantly reduced the elongation and stress at failure (respectively 34% and 21% reduction for extruded UHMWPE compared with 20% and 7% reduction for extruded LDPE).

### 3.2. Static and Dynamic Compression

Static compression experiments of LDPE and UHMWPE were performed on a universal material-testing machine (INSTRON 5969) and the dynamic experiments were performed on an SHPB apparatus. Variations in strain rates during the SHPB experiment significantly affected the resultant stress–strain data. Therefore, to obtain a family of dynamic true stress-strain curves of LDPE and UHMWPE at the same strain-rate, the geometrical dimensions and loading pressures were carefully controlled. Table 1 shows that the average error in strain-rate was less than 90 s^−1^. The main purposes of static and dynamic compression tests were to obtain comparative properties for compression of the two polyethylenes, to measure the effect of strain-rate sensitivity, and to develop a rate-dependent constitutive model that represents yielding and plastic flow of extruded polyethylene.

**Figure 11:**
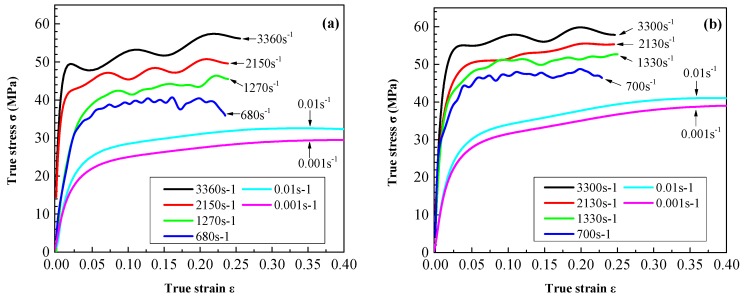
Typical compressive true stress-strain curves of (**a**) LDPE and (**b**) UHMWPE under a wide range of strain-rates.

Typical compressive true stress–true strain curves for LDPE and UHMWPE at six different strain-rates and room temperature are shown in Figure 11. These curves show similar characteristics of yielding and plastic flow. These features are similar to those reported previously for many polymers [9,23,24]. It is well known that strain hardening of polyethylene can be attributed to both the molecular structure and crystalline formation [9]. Furthermore, we see that both LDPE and UHMWPE specimens are sensitive to strain-rate. The yield stress and steady flow stress increase substantially with increasing strain rate. For example, at a specific offset strain of 2%, the LDPE and UHMWPE specimens recorded proportional limits of approximately 14.98 and 19.63, 20.37 MPa and 23.46 MPa for strain rates of 0.001 and 0.01 s^−1^, respectively. Figure 11 shows that the initial flow stress increased rapidly as the true strain increased to 0.1 for each of the stress-strain curves. However, when true strains exceed 0.1, the flow stress increased only slightly with increasing strain. By comparing Figure 11a,b, it is observed that for small strain-rates, the strain-hardening of UHMWPE is larger than that of LDPE.

**Figure 12:**
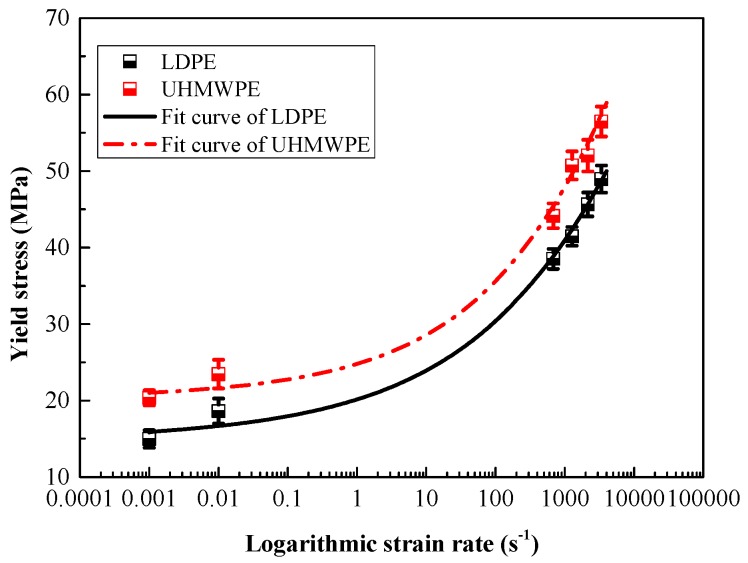
Yield stress of LDPE and UHMWPE under wide range of strain rates (from 0.001 to 3300 s^−1^).

Compression yield stresses of LDPE and UHMWPE under a wide range of strain-rates were compared in Figure 12. The yield stresses of both LDPE and UHMWPE at high strain-rates were much larger than at low strain-rates. Moreover, at a given strain rate, the yield stress of extruded UHMWPE was larger than that of LDPE—at least 17% larger. Since the polymer chains of UHMWPE had few branch points, there are more long-chain molecules at the same density than LDPE, most of those were required to support the applied stress at a certain strain, the applied stress to squeeze and deform the molecular structure was larger than that of LDPE.

However, the branched nature of the side groups that exist in LDPE molecular chains form more spaces and gaps in the molecular structure. These spaces and gaps form weak points that tend to reduce the yield stress. Meanwhile, for all polyethylene specimens that were tested, the compression modulus increased with increasing strain-rate. At high strain-rates, the motion of the molecular structures was limited by inertia. Moreover, the compression modulus of UHMWPE is higher than that of LDPE (Table 4). It means that at same strain-rate, the UHMWPE will resist a higher stress at same strain before reach its yielding limit.

### 3.3. Constitutive Equation

Generally, a material’s plastic flow stress $\sigma_{pl}$ can be expressed as [25]:

(4) $$\sigma_{pl}=f(\varepsilon_{pl},T)\cdot R(\dot{\varepsilon }),$$

where $f(\varepsilon_{pl},T)$ is the static stress-strain behavior, $R(\dot{\varepsilon })$ is the ratio of the yield stress at nonzero strain-rate to the static yield stress.

In the past, a variety of numerical models have been proposed to represent the material behavior of extruded polyethylene; these include classical J_2_-plasticity model [26], “Arruda–Boyce” model [27,28], “hybrid” model [23], modified Eyring equation [9], *etc*. However, none of these models can describe the non-linear deformation behavior perfectly, when we consider the effects of strain-rate sensitivity, strain-softening and strain-hardening.

In this investigation, we aim to obtain the material properties required to predict the yield behavior of the polyethylene specimens subject to high-speed impact. For simplicity and practicability in engineering applications, the true stress-strain curves obtained from experimental data can be imported into Abaqus directly [6]. In view that the yield stress and the logarithm of strain rate is showing a strong exponential relationship in Figure 12 the Cowper–Symonds (C-S) model was applied to incorporate the strain-rate effect [24,29]. The existing Cowper–Symonds model can be written as:

(5) $${\dot{\varepsilon }}_{pl}=D{\left(R-1\right)}^{p},$$

where *D* and *p* are material parameters to be determined from experimental observations, and $\dot{\varepsilon }$ is the strain-rate. The compression experimental data for a small strain-rate of 0.001 s^−1^ was selected as the static value; Cowper–Symonds assumes that the yield stress $\sigma_{s}$ at a high strain-rate has a power-law relationship to the static yield stress $\sigma_{0}$:

(6) $$\sigma_{s}=\sigma_{0}[1+{\left(\frac{{\dot{\varepsilon }}_{pl}}{D}\right)}^{1/p}],$$

directly taking the logarithm of both sides of Equation (5) without regard for positive or negative values of the function:

(7) $$\log_{10}{\dot{\varepsilon }}_{pl}=log_{10}D+p\;\log_{10}(R-1).$$

**Figure 13:**
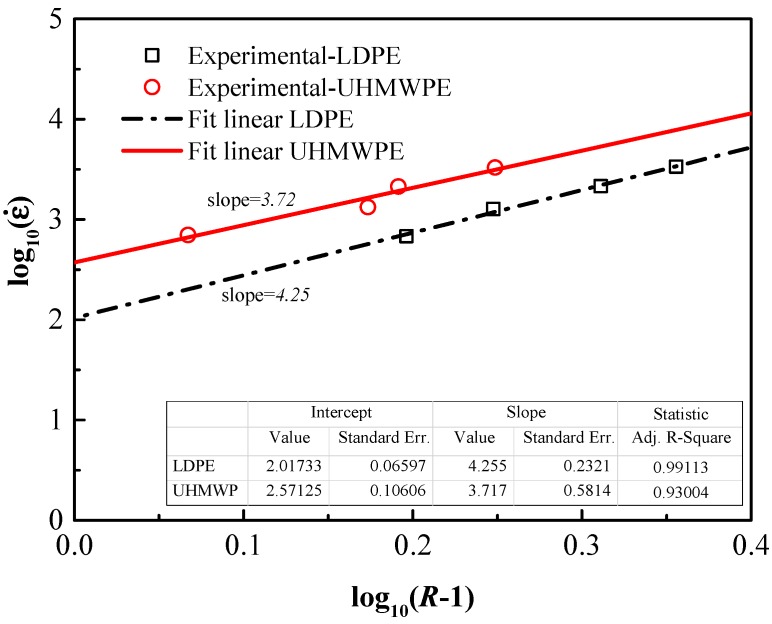
Master curves of the form $\log_{10}\dot{\varepsilon }$ *versus* $\log_{10}(R-1)$ obtained with LDPE and UHMWPE tests at different strain rates. The fit lines indicate the coefficients of C-S model.

This results in $\log_{10}{\dot{\varepsilon }}_{pl}$ as a linear function of $\log_{10}(R-1)$. Figure 13 shows the master linear curves of the form $\log_{10}{\dot{\varepsilon }}_{pl}$ *vs.* $\log_{10}(R-1)$ for LDPE and UHMWPE obtained from tests at different strain-rates. In the fitted regression lines, the intercepts represent the value of $\log_{10}D$, and *p =* slope*.* The coefficients *D* and *p* for C-S models were obtained from Figure 13, and are summarized in Table 5.

Quantitatively, Equation (7) is valid within experimental error as R-squared values are approximately 99% and 93% for LDPE and UHMWPE. Based on the calculated errors, we find that the C-S model is acceptable to predict the strain-rate effect on the flow stress of the polyethylene specimens under a wide range of strain rates.

It was found that, while *p* is nearly constant and equal to 4.26 and 3.72 for UHMWPE and LDPE respectively, the coefficients *D* is 104 and 372 for UHMWPE and LDPE. It can be inferred from the fit lines that the strain rate hardening effect of LDPE is more sensitive to strain-rates for small strain rates, while that of UHMWPE is more sensitive to strain-rates at high strain-rates. Consequently, UHMWPE will have a better performance than LDPE under high strain-rate deformation.

## 4. Conclusions

This investigation performed tensile and compression material tests at quasi-static strain rates of 0.001 and 0.01 s^−1^ on two types of polyethylene. In addition, dynamic impact testing was carried out using a split Hopkinson pressure bar (SHPB) to achieve strain rates up to 3300 s^−1^. The effect of strain-hardening and strain-rate hardening of two types of polyethylene specimens were measured under both tension and compression loads. The difference in molecular chain structure for the two polyethythenes can account for the differences in mechanical properties. The following conclusions were obtained from the present investigation:

Because of similarity in the density of molecular chains but differences in their length and branching, extruded LDPE and extruded UHMWPE had similar isotropic properties in the small strain elastic region, but different behaviors in the large strain, plastic flow region.

At quasi-static rates of strain, extruded UHMWPE had more strain-hardening than extruded LDPE, together with a very large elongation at failure.

Comparing compression tests at strain rates from 0.001 up to 3300 s^−1^, both LDPE and UHMWPE specimens had increasing yield stress with increasing strain rate. At all strain rates, the compression yield stress of extruded UHMWPE was at least 17% larger than that of extruded LDPE.

The Cowper–Symonds model was adopted to represent the effect of strain-rate on yield stress. The C-S parameters obtained are in good agreement with the experimental results, as R-squared values of fit lines are approximately 99% and 93% for LDPE and UHMWPE, respectively.

The C-S model shows that the strain-rate hardening effect of UHMWPE was more sensitive to strain-rate for higher strain rates, and that of LDPE was more sensitive for smaller strain rates. This indicates that at the high strain-rates that are representative of impact on armor, UHMWPE will perform better than LDPE.

Despite defects, non-uniformity of mixing and plastination, the well-regulated arrangement and long molecular chains of extruded polyethylene significantly improve its mechanical properties. Extruded UHMWPE is more amenable to large deformation and high-speed impact conditions than LDPE because of its long molecular chains that are relatively seldom branched.

## Figures and Tables

**Table 1:** Material properties.

Type	*ρ* (Kg/m^3^)	*E* (MPa)	σ*_y_* (MPa)	ε (At break)	*T* (°C, Vicat softening point)
LDPE	920	750	≥19	≥3	80
UHMWPE	950	800	≥20	≥4.5	79

**Table 2:** Thickness of the specimens and load pressure.

Materials	Thickness (mm)	Load pressure (kPa)	Average strain rates (s^−1^)	Max. deviation (s^−1^)
LDPE	4	405	3,360	±80
	5	304	2,150	±30
	5	152	1,270	±40
	10	152	680	±30
UHMWPE	4	405	3,300	±90
	5	304	2,150	±40
	5	152	1,330	±70
	10	152	700	±30

**Table 3:** Summary of quasi-static tensile test results for LDPE and UHMWPE. Averages of duplicate tests are shown below.

Material	Strain rates (s^−1^)	Angle	Tensile stress (MPa)	Young’s modulus (MPa)	Engineering stress at break (MPa)	Engineering strain at break
LDPE	0.001	0°	41.07 ± 0.70	870.6 ± 10.7	30.36 ± 0.57	5.84 ± 0.79
		45°	40.19 ± 0.91	839.9 ± 25.2	30.89 ± 0.97	6.21 ± 0.78
		90°	40.43 ± 0.32	884.4 ± 18.4	31.23 ± 0.63	5.90 ± 0.95
	0.01	0°	41.29 ± 0.14	905.5 ± 23.5	27.98 ± 0.83	4.67 ± 0.43
UHMWPE	0.001	0°	40.90 ± 1.19	912.5 ± 32.1	43.19 ± 0.52	10.12 ± 0.84
		45°	38.14 ± 0.23	896.8 ± 25.2	41.32 ± 0.91	9.23 ± 0.91
		90°	38.77 ± 0.78	814.3 ± 15.5	41.71 ± 0.34	9.45 ± 0.52
	0.01	0°	41.53 ± 0.65	922.5 ± 21.2	34.07 ± 0.97	6.62 ± 1.08

**Table 4:** Summary of compression test results for LDPE and UHMWPE for a range of strain-rates. Averages of multiple tests are shown below.

Material	Strain-rates (s^−1^)	Offset yield strength σ_0.2_ (MPa)	Compression modulus (GPa)
LDPE	0.001	14.98 ± 1.15	0.722 ± 0.025
	0.01	19.63 ± 1.63	0.904 ± 0.034
	680	38.52 ± 1.31	1.797 ± 0.246
	1270	41.47 ± 1.22	2.227 ± 0.377
	2150	45.64 ± 1.56	2.267 ± 0.671
	3360	48.96 ± 1.77	2.428 ± 0.674
UHMWPE	0.001	20.37 ± 0.91	0.905 ± 0.027
	0.01	23.46 ± 1.87	1.150 ± 0.066
	700	44.15 ± 1.62	2.489 ± 0.752
	1330	50.74 ± 1.84	2.555 ± 0.673
	2130	52.04 ± 2.08	2.737 ± 0.358
	3300	56.48 ± 1.95	2.970 ± 0.737

**Table 5:** Coefficients for Cowper–Symonds model.

Material	*D*	*p*
LDPE	104	4.26
UHMWPE	372	3.72
